# Nanomaterials-Based Sensing Strategies for Electrochemical Detection of MicroRNAs

**DOI:** 10.3390/ma7075366

**Published:** 2014-07-23

**Authors:** Ning Xia, Liping Zhang

**Affiliations:** College of Chemistry and Chemical Engineering, Anyang Normal University, Anyang 455000, China; E-Mail: lpzhang@aynu.edu.cn

**Keywords:** nanomaterials, miRNAs, electrochemistry, biosensors

## Abstract

MicroRNAs (miRNAs) play important functions in post-transcriptional regulation of gene expression. They have been regarded as reliable molecular biomarkers for many diseases including cancer. However, the content of miRNAs in cells can be low down to a few molecules per cell. Thus, highly sensitive analytical methods for miRNAs detection are desired. Recently, electrochemical biosensors have held great promise as devices suitable for point-of-care diagnostics and multiplexed platforms for fast, simple and low-cost nucleic acid analysis. Signal amplification by nanomaterials is one of the most popular strategies for developing ultrasensitive assay methods. This review surveys the latest achievements in the use of nanomaterials to detect miRNAs with a focus on electrochemical techniques.

## 1. Introduction

MicroRNAs (miRNAs) are single-strand non-coding RNA molecules typically containing 18–25 nucleotides. They play important functions in numbers of biological processes, such as developmental regulation, proliferation, differentiation, cardiogenesis, and epigenetic inheritance [1,2,3]. Expression levels of miRNAs may provide useful diagnostic and prognostic implications since their aberrant expression has been correlated with cancer (prostate, breast, colon, lung,* etc.*) and other diseases (diabetes, heart diseases,* etc.*) [4,5]. However, the short length, high sequence similarity and low abundance (down to a few molecules per cell) of miRNAs impose difficulty to developing the detection methods [6,7,8,9]. Recently, electrochemical biosensors have held great promise as devices suitable for point-of-care diagnostics and multiplexed platforms for fast, simple and low-cost nucleic acid analysis [10,11,12]. Currently, the increasing demand for measuring the ultralow levels of miRNAs with electrochemical techniques is driving the enhancement of sensitivity. Typically, signal amplification by nanomaterials is one of the most popular strategies for developing ultrasensitive electrochemical analytical methods. The progress in the development of nanomaterials-based electrochemical miRNAs biosensors are addressed in this work.

## 2. Nanomaterials-Based Electrochemical Strategies for miRNAs Detection

To date, nanomaterials used for amplified electrochemical bioassays of miRNAs include metallic/magnetic nanoparticles, carbon-based nanostructures, quantum dots, and nanostructured electrodes. Their preparation, modification and sensing principle were presented herein.

### 2.1. Metallic Oxide

**Figure 1:**
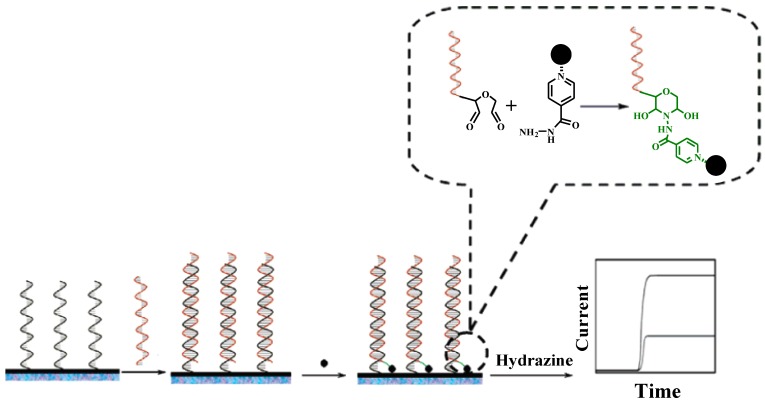
Schematic illustration of miRNA assay using electrocatalytic OsO_2_ nanoparticles. Reprinted with permission from [13]. Copyright 2006 American Chemical Society.

The first electrochemical miRNAs biosensor was reported by Gao* et al.* [13] in 2006. In the work, electrocatalytic OsO_2_ nanoparticles (~25 nm) modified with isoniazid were employed for signal amplification. In the detection system, miRNAs were pre-oxidized with periodate to generate 2′- and 3′-terminal dialdehydes at the 3′ end (Figure 1). After the capture of the oxidized miRNAs by the DNA probes immobilized on the electrode, the OsO_2_ nanoparticles were then anchored via the interaction of isoniazid and dialdehyde, leading to the production of a catalytic current by oxidization of hydrazine. The detection limit of this method was 80 fM with a detectable miRNAs concentration up to 0.2 nM.

**Figure 2:**
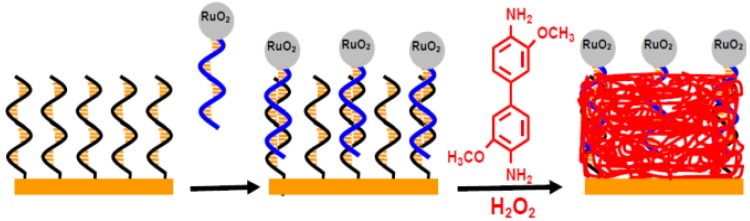
Schematic illustration of the miRNAs biosensor based on RuO_2_ NPs-catalyzed miRNAs-templated deposition of a thin PBD insulating film. Reprinted with permission from [19]. Copyright 2011 American Chemical Society.

Nucleic acid can guide the enzymatic deposition of nanoparticles and nanowires where the phosphate groups serve as templates [14,15]. Gao’s group also developed simple and sensitive electrical miRNAs biosensors by the miRNAs-guided deposition of polyaniline (PAn) nanowires or insulating poly(3,3′-dimethoxybenzidine) (PDB) polymer film with enzymatic polymerization methods [15,16,17]. In the methods, the electrostatic interaction of phosphate group and aniline or 3,3′-dimethoxybenzidine (DB) is responsible for guiding the formation of nanowires or film. Similarly, they found that ruthenium oxide nanoparticles (RuO_2_ NPs) can also catalyze the polymerization of DB and aniline [18,19]. In 2010, they described a miRNAs biosensor based on RuO_2_ NPs-guided formation of PAn nanowires in the presence of H_2_O_2_. In the work, gold electrodes covered with the mixed monolayers of peptide nucleic acid (PNA) and 4-mercaptoaniline (MAn) were used for the capture of miRNAs [18]. The neutral PNA backbone can alleviate the electrostatic absorption of cationic aniline on the sensing surface, thus producing a high signal/noise ratio. A detection limit of 2 fM and a dynamic range of 5 fM~2 pM were achieved by monitoring the current of PAn with square wave voltammetry. Lately, they reported a miRNAs biosensor based on RuO_2_ NPs-initiated polymerization of DB on gold electrodes covered with the mixed monolayers of DNA capture probes (CPs) and MAn (Figure 2) [19]. Hybridization with RuO_2_-tagged miRNAs and incubation in a mixture of DB/H_2_O_2_ led to the formation of an insulating PDB film and the increase in the electrochemical impedance. The amount and insulating capability of the deposited PDB correlated to the miRNAs concentration in the range of 6 fM to 2 pM. After incubating the sensing electrode in the mixed DB/H_2_O_2_ solution for 60 min, a detection limit of 3 fM was obtained by electrochemical impedance spectroscopy. In the work, RuO_2_ NPs were prepared by dissolving RuCl_3_ in a solution of 1-*n*-butyl-3-methylimidazolium hexafluorophosphate, heating at 50–60 °C and stirring under argon [20]. The average diameter of the NPs was determined to be 2.4 ± 0.5 nm. In these two detection systems, the hybridized miRNAs strand and the tagged RuO_2_ NP act as the template and catalyst respectively for the deposition of PAn or PDB. Moreover, the miRNAs were pre-oxidized by sodium periodate to form 2′- and 3′-terminal dialdehydes at the 3′ end of the molecules and then enriched by 4-(2-aminoethyl) pyridine (AEP)-coated RuO_2_ NPs through the formation of an imine bond between dialdehyde on the miRNAs and amine on the RuO_2_ NPs.

### 2.2. Gold Nanoparticles

**Figure 3:**
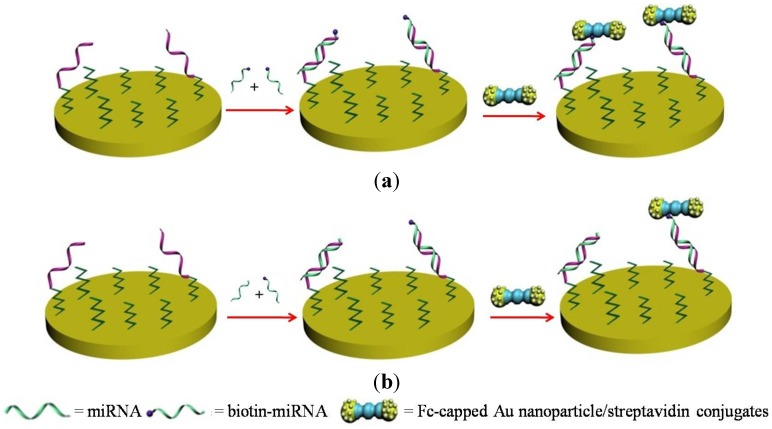
Schematic representation of the miRNA detection. The absence of a miRNA with the same sequence as that of the externally added biotin-miRNA leads to more Fc-capped gold nanoparticle/streptavidin conjugates attached to the electrode and a large voltammetric signal (**a**). In the presence of the miRNA, a smaller number of the conjugates are attached to the electrode due to the competitive hybridization reaction and consequently a lower voltammetric signal is produced (**b**). Reprinted with permission from [25]. Copyright 2012 American Chemical Society.

Gold nanoparticles (AuNPs) as labels have been widely used in diagnostics and detection because of their unique characteristics, such as high surface-to-volume ratio, high surface energy, ability to decrease proteins-metal particles distance, and the functioning as electron conducting pathways between prosthetic groups and the electrode surface [21,22,23,24]. Typically, AuNPs are labeled with biorecognition elements for the recognition of targets and with response molecules (redox tags or enzymes) for the signal readout. Thus, the capture of AuNPs on electrode surface can generate numbers of electroactive molecules and induce a great change in the current. For example, in view of the well-defined and reversible redox reaction of ferrocene (Fc), Wang* et al.* [25] performed the voltammetric detection of miRNAs using Fc-capped Au nanoparticle/streptavidin conjugates prepared by mixing 6-ferrocenylhexanethiol dissolved in hexane and AuNPs/streptavidin conjugates dispersed in PBS buffer (Figure 3). In this work, biotinylated miRNAs (biotin-miRNAs) whose sequence is the same as that of the miRNAs target were allowed to compete with the miRNAs targets for binding DNA probes that were pre-immobilized onto the electrode. Voltammetric determination of the miRNAs was achieved by introducing Fc-capped Au nanoparticle/streptavidin conjugates onto the sensing surface via the biotin-streptavidin interaction. The oxidation current of Fc was found to be inversely proportional to the concentration of target miRNAs. Because of the signal amplification of AuNPs, miRNAs at the concentration of 10 fM could be determined readily. The relative standard deviations (RSDs) are all below 5% in the concentration range of 1 fM to 2 pM. Although the method is sensitive, regenerable and reliable, it requires the use of biotin-labeled miRNAs and streptavidin-conjugated AuNPs. The main difference in the structure of RNA* versus* DNA is the presence of a hydroxyl group at the 2′ position of the ribose sugar in RNA, which makes the RNA molecule contain cis-diol at the end of the chain and enables miRNAs to be distinguished from DNA. Hydrophilic phenylboronic acid can form covalent bond with cis-diol [26,27,28]. The formation of borate ester between phenylboronic acid and ribose sugar allows for the capture, separation and immobilization of nucleotides and RNA by boronic acid-functionalized materials [29,30,31,32]. For these views, we developed a label-free and sensitive method for miRNAs detection based on the dual-amplification of 4-mercaptophenylboronic acid (MBA)-capped AuNPs (MBA-AuNPs) and dopamine (DA)-capped AuNPs (DA-AuNPs) [33]. Specifically, miRNAs were captured by the pre-immobilized DNA probes and then derivatized with MBA-AuNPs through the formation of tight covalent bonds between phenylboronic acid moieties on MBA-AuNPs and diol groups of miRNAs. The MBA-AuNPs allowed for the capture of DA-AuNPs by the interaction between DA and phenylboronic acid, thus facilitating the amplified voltammetric detection of miRNAs at the concentration ranging from 0.1 to10 pM. The detection limit was estimated to be 45 fM.

In view of the high surface-to-volume ratio and electro-catalytic performance of AuNPs, signal amplification by AuNPs may follow two strategies: one in which AuNPs were modified on electrode for the immobilization of receptors and another in which AuNPs were used to load recognition elements and a large number of reporters. Hemin as an active cofactor for many enzymes can catalyze the peroxidation reaction. When incubation with guanine-rich DNA aptamer, hemin can intercalate into a G-quadruplex structure to form a hemin/G-quadruplex DNAzyme with peroxidase-like ability [34,35,36]. Ai and co-workers reported a signal-amplified electrochemical miRNAs biosensor by employing hemin-G-quadruplex as the signal unit [37]. In the work, AuNPs-modified gold electrodes covered with hairpin-structured DNA probes were employed for the construction of the biosensor. Hybridization of target miRNAs to the probes opened the hairpin structure, thus facilitating the capture of AuNPs that were loaded with the hemin aptamer and the DNA sequence complementary partly to the DNA probe. The electrochemical signal resulting from the hemin-G-quadruplex was collected by chronoamperometry. A detection limit of 3.96 pM and a dynamic range of 5 pM–5 nM were obtained. Furthermore, they found that the hemin-G-quadruplex on AuNPs can significantly promote the catalysis of H_2_O_2_ by oxidation of hydroquinone; this led to an obvious reduction current of benzoquinone [38]. This method could be used to detect miRNAs with a detection limit of 6 fM and a dynamic range of 0.01–500 pM. Based on the intrinsic peroxidase-like activity of hemin, they also developed a label-free method for miRNAs detection by employing carboxylic graphene-hemin hybrid nanosheets instead of hemin-G-quadruplex on AuNPs-modified glass carbon (GC) electrode [39]. As a result, the detection limit of 0.17 pM is higher than that achieved by the hemin-G-quadruplex.

Enzyme amplification was one of the most commonly employed strategies to enhance detection sensitivity in electrochemical bioassays. However, single amplification by enzyme label is not sufficient for detecting an ultra-low number of miRNAs [40,41,42,43]. For example, simple enzyme-amplified electrochemical genosensor where the esterase-oligonucleotide conjugate is used as the recognition unit to produce an electrochemical signal typically obtains a detection limit of 2 pM [41]. In view of the high surface-to-volume ratio of AuNPs, multienzyme report probes are prepared by bioconjugating large amounts of enzymes on AuNPs for signal amplification [44,45]. For example, Yin* et al.* [44] reported the amplified detection of miRNAs on AuNPs/graphene-modified electrodes with locked nucleic acid (LNA) as the capture probe. AuNPs loading with the reported DNA and streptavidin-horseradish peroxidase (SA–HRP) were brought to the electrode via the hybridization of target miRNAs and DNA, which catalyzed the chemical oxidation of hydroquinone by H_2_O_2_. As a result, a detection limit of 6 fM and a linear range of 0.01–700 pM were achieved. The detection limit is lower than that (0.4 pM) obtained by the single amplification of HRP [46].

**Figure 4:**
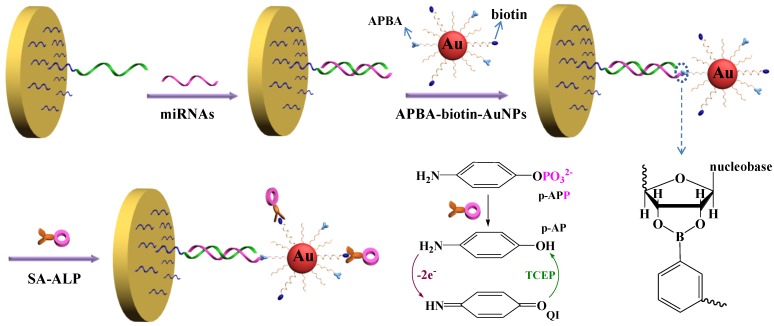
Schematic representation of the label-free detection of miRNAs based on the triple signal amplification of APBA-biotin-AuNPs, SA-ALP and the *p*-AP redox-cycling reaction. Reprinted with permission from [48]. Copyright 2014 Elsevier.

Alkaline phosphatase (ALP), one of the most used enzymatic labels for design of absorption biosensors, can remove a phosphate group from the substrate to produce an electroactive species. For example, it dephosphorylates p-aminophenyl phosphate (*p*-APP) enzymatically to produce *p*-aminophenol (*p*-AP), which is readily detected on electrode. However, *p*-AP-based analytical methods suffer from drawback related to its limited stability. To overcome this defect, reducing reagents can be added to the reaction mixture to prevent *p*-AP oxidation. Moreover, the reducing reagents can regenerate *p*-AP from p-quinone imine (QI, the oxidation production of *p*-AP) in the electrochemical detection. The process of *p*-AP regeneration is called *p*-AP redox cycling [47]. We recently provided a highly sensitive method for miRNAs detection with the triple signal amplification of AuNPs, ALP and *p*-AP redox cycling (Figure 4) [48]. The label-free strategy is also based on the differences in the structure of RNA and DNA. Specifically, miRNAs captured by the pre-immobilized DNA probes allowed 3-aminophenylboronicacid (APBA)/biotin-modified multifunctional AuNPs (APBA-biotin-AuNPs) to be attached through the formation of boronate ester covalent bonds. Then, streptavidin-conjugated ALP (SA-ALP) was anchored on the electrode via the strong biotin-streptavidin interaction. After the addition of the *p*-APP substrate, the enzymatic conversion from *p*-APP to *p*-AP proceeded. The produced *p*-AP could be cycled by a reducing reagent after its electro-oxidization, thus enabling an increase in its anodic current. The results indicated that the current increased linearly with miRNAs concentrations in the range of 10 fM~5 pM. The detection limit of this method was calculated to be 3 fM.

### 2.3. Silver Nanoclusters

**Figure 5:**
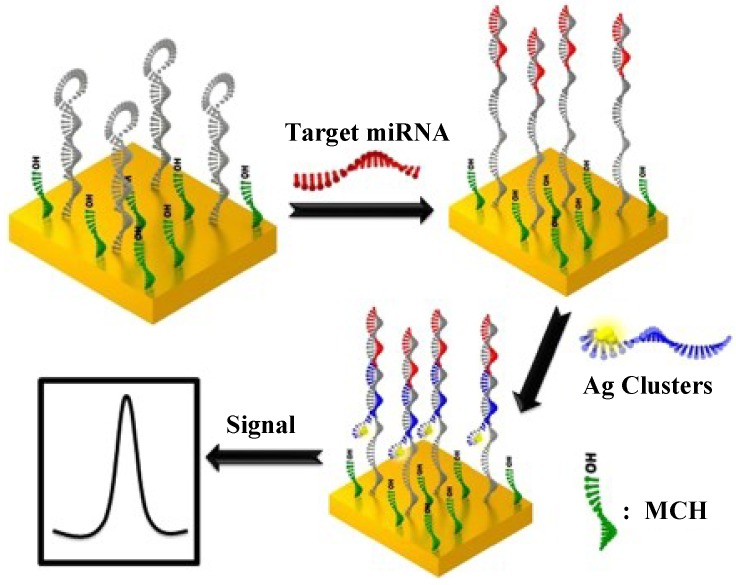
Illustration of electrochemical detection of miRNA using oligonucleotide encapsulated Ag-NCs. Reprinted with permission from [50]. Copyright 2012 American Chemical Society.

The use of metal nanoparticles (e.g., Ag, Pd) as catalysts is analogous to the way of enzymes and might overcome some of the problems related to the inherent thermal and environmental instability of the enzyme [49,50]. For example, silver nanoclusters (Ag-NCs) display efficient catalytic property toward H_2_O_2_ reduction. Dong* et al.* [50] presented a simple, sensitive and label-free method for miRNAs detection using DNA encapsulated Ag-NCs as the electrochemical probes (Figure 5). In the work, the Ag-NCs were synthesized by adding the template DNA strand and AgNO_3_ to a citrate buffer with a Ag^+^/DNA concentration ratio of 8:1. Fresh NaBH_4_ solution was then added to the mixed solution in ice bath. After shaking for 1 min, the spherical Ag-NCs with a mean diameter of approximate 2 nm were produced. The hybridization between the target miRNAs and the molecular beacon (MB) probes allowed for the introduction of Ag-NCs-functionalized probes onto the electrode. Then, an electrochemical signal responsive to H_2_O_2_ reduction was observed. The detection limit was 67 fM with a linear current-concentration relationship in the range of 10 nM~100 fM.

### 2.4. Carbon Nanomaterials

**Figure 6:**
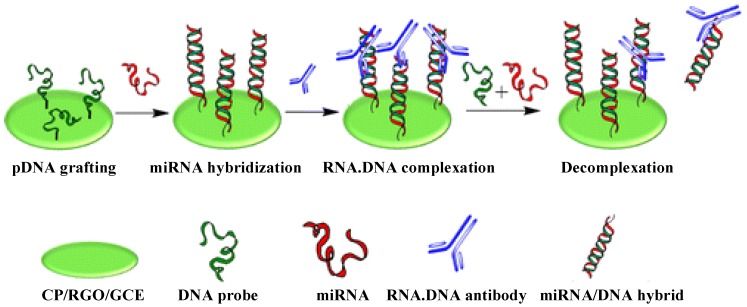
Schematic of miRNAs detection principle based on antibodies directed to RNA/DNA hybrids using graphene-composite electrodes. Reprinted with permission from [55]. Copyright 2013 American Chemical Society.

Carbon nanomaterials are frequently incorporated as sensing elements since they offer unique advantages that span several domains, such as a high surface-to-volume ratio, high electrical conductivity, chemical stability, biocompatibility, and robust mechanical strength [51,52,53]. Recently, carbon nanotubes (CNTs) have been applied in the electrochemical sensing of miRNAs due to their metallic, semi-conducting and superconducting electron-transport abilities and large capacity to be loaded with various biomolecules. Pham’s group reported a label-free and reagentless miRNAs biosensor based on an interpenetrated network of multi-walled CNTs (MWCNTs) and electroactive polymer [54]. The specific NH_2_-modified oligonucleotide probes were grafted onto the glassy carbon electrode that were coated with MWCNTs and poly-[5-hydroxy-1,4-naphthoquinone]-copolymer-[3-(5-hydroxy-1,4-dioxo-1,4-dihydronaphthalen-2(3)-yl) propanoic acid] (poly(JUG-co-JUGA). The quinine group embedded in the backbone of poly(JUG-co-JUGA) endowed the nanostructured polymer film well-defined electroactivity in neutral aqueous medium. The enhancement of the polymer electroactivity by specific hybridization of target miRNAs induced an increase in the current. The detection limit of this method was calculated to be 8 fM. Moreover, they also reported a simple, sensitive and label-free immunosensor for miRNAs detection on a conducting polymer/reduced graphene oxide-modified electrode (Figure 6) [55]. In the work, the water-dispersed reduced graphene oxide (RGO) was synthesized by reducing graphene oxide (GO) with epigallocatechin gallate (EGCG) at 80 °C. The conducting polymer-coated RGO-modified GC electrode (CP/RGO/GCE) was prepared by electropolymerizating 5-hydroxy-1,4-naphthoquinone (JUG) and 3-(5-Hydroxy-1,4-dioxo-1,4-dihydronaphthalen-2(3)-yl) propanoic acid (JUGA) on RGO-modified GC electrode in the potential cycling range of 0.4–1.1 V (*versus* saturated calomel electrode) for 25 cycles at 50 mV∙s^−1^. Because of the strong steric hindrance, DNA probes closely packed on the electrode surface decreased the diffusion coefficient of counterions. Conversely, DNA/RNA hybridization caused conformational reorganization of the double strands, which created a free space on the electrode surface and induced a significant current increase. The method could be used to detect miRNAs in the concentration range of 1 fM to 1 nM with a detection limit of 5 fM. Additionally, based on the simple steric hindrance principle, capture of RNA-DNA antibodies induced a current decrease. Thus, the “Off” signal could be returned to an “On” signal when RNA/DNA hybrids were introduced into the solution.

**Figure 7:**
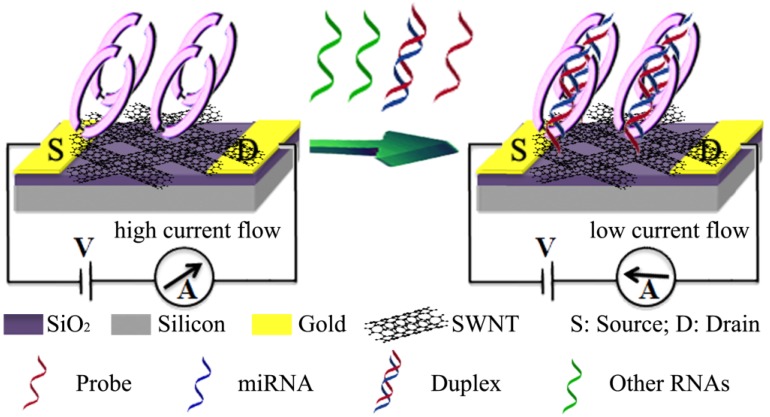
Schematic of miRNA detection principle by the p-19 functionalized CNTs-FET nanobiosensor. Reprinted with permission from [59]. Copyright 2013 American Chemical Society.

Field-effect transistor (FET) is one of promising techniques to develop label-free, rapid and sensitive electrochemical biosensors [56,57]. p19 protein, a 19 kDa protein expressed by plant viruses, functions as a suppressor of the RNA silencing pathway and binds with high affinity only to double stranded/duplex RNA (dsRNA) in a size-specific and sequence-independent manner [58]. Recently, Ramnani* et al.* [59] reported a new electrochemical miRNAs nanobiosensor using a p19-functionalized CNTs FET (Figure 7). It started with the construction of the CNTs-FET device using 3-aminopropyltriethoxysilane (APTES)-assisted assembly of CNTs from a 95% enriched semiconductive nanotubes ink across 3 μm spaced microfabricated interdigitated gold electrodes acting as source and drain on Si/SiO_2_ followed by annealing in air at 250 °C for an hour [59]. Interaction of dsRNA and p19 induced the conductance change of CNTs. The target miRNAs in the concentration range from 1 aM to 10 fM could be detected readily in the presence of a million fold excess of total RNA and other miRNAs.

The oxidation of guanine in RNA can generate a voltammetric signal. CNTs exhibit excellent electrochemical property for guanine oxidation [60,61]. For these views, Li* et al.* [62] developed a simple and label-free electrochemical biosensor for miRNAs detection by measuring the oxidation current of guanine on a MWCNT-modified GC electrode. The specific DNA probes were immobilized onto the MWCNT-modified electrode for the capture of miRNAs. The detection limit of 1 pM was lower than that (5 nM) obtained by the direct oxidation of guanine in the RNA/DNA hybrids on a screen-printed electrode [63].

### 2.5. Quantum Dots

**Figure 8:**
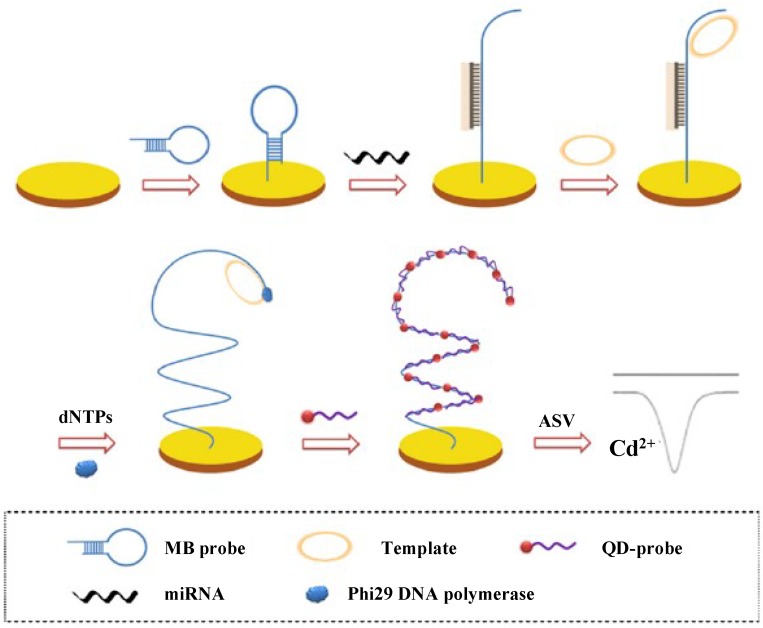
Schematic representation of the designed strategy for miRNAs detection. Reprinted with permission from [65]. Copyright 2013 Elsevier.

Quantum dots (QDs) have been widely employed as electroactive labels for the detection of protein and DNA in view of their unique amplification features [64]. Recently, QDs have also been involved in the detection of miRNAs. For example, Wang* et al.* [65] developed an ultrasensitive CdS QDs-based method to detect miRNAs by employing molecular beacon (MB) structure guided rolling circle amplification (RCA) (Figure 8). Upon hybridization with the target miRNAs, the LNA-MB CPs opened to release the RCA primers. The resulting RCA product containing thousands of repeated DNA sequences allowed for the periodic hybridization with the QDs-tagged detection probes. The Cd^2+^ ions released from the CdS QDs were then determined by anodic stripping voltammetry. The detection limit of this method was 0.32 aM with a six-decade dynamic range. Furthermore, Zhu* et al.* [66] developed a label-free and PCR-free electrochemical biosensor for multiplexed assay of miRNAs based on the combination of the high base-mismatch selectivity of ligase chain reaction (LCR) and the remarkable voltammetric signature of electrochemical QDs barcodes. In the work, PbS and CdS QDs were labeled with the report probes of RP1 and RP2, respectively, to form the PbS-RP1 and CdS-RP2 conjugates. The CP1 and CP2 CPs were co-immobilized on the magnetic bead to produce the bead-CP1CP2 conjugate. Then, miRNAs were incubated with the bead-CP1CP2, PbS-RP1 and CdS-RP2 conjugates, followed by the addition of T4 DNAligase. After dissociation of the QDs barcodes from the magnetic bead, two target miRNAs can be simultaneously determined with linear ranges of 50 fM~30 pM and 50 fM~1050 pM and detection limits of 12 fM and 31 fM, respectively.

### 2.6. Nanostructured Electrodes

**Figure 9:**
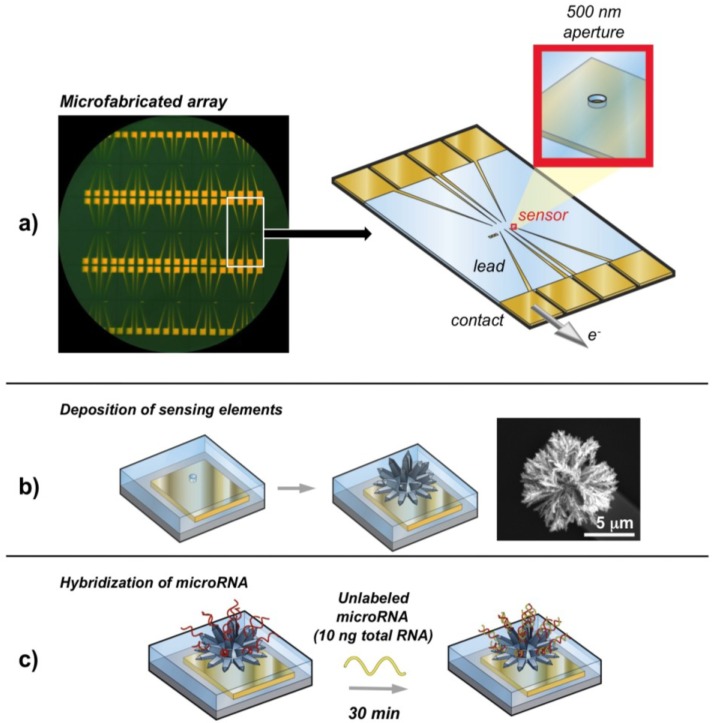
Electronic microRNAs detection with nanostructured microelectrode (NME) chips. (**a**) Photograph (**left**) showing microfabricated chips that feature 500 nm openings for the electrochemical deposition of NMEs, and illustration (**right**) of the chip structure; (**b**) Schematic illustration of the generation of sensing elements by palladium electrodeposition. A scanning electron microscope image of a deposited nanostructured microelectrode is shown on the right; (**c**) Hybridization of unlabeled microRNA in samples containing 10 ng of total RNA to a probe-modified chip. After 30 min, hybridization can be read out electrochemically by using an electrocatalytic Ru^III^/Fe^III^ reporter system. Reprinted with permission from [67]. Copyright 2009 John Wiley and Sons.

**Figure 10:**
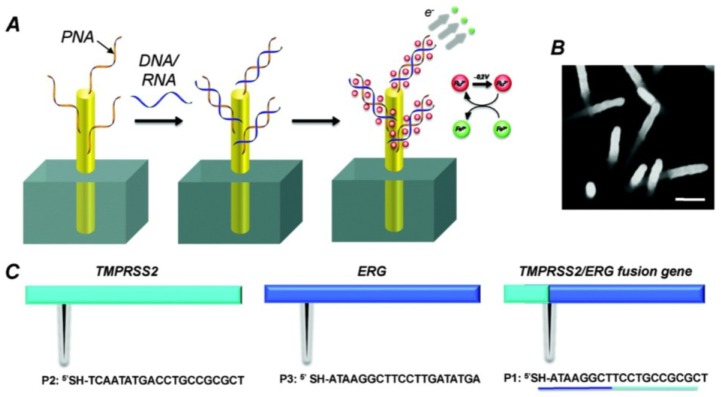
(**A**) Schematic illustration of electrocatalytic detection of nucleic acids using nanowire sensors; (**B**) Representative scanning electron micrograph (SEM) of gold nanowires used in this Study; and (**C**) Sequences of probes complementary to TMPRSS2 (P2), ERG (P3), or the TMPRSS2:ERG fusion (P1). Reprinted with permission from [70]. Copyright 2009 American Chemical Society.

Nanoparticles-modified electrodes can increase the surface area and numbers of binding events. Growth of nanoparticles by electrodeposition is especially attractive since it guides the direct formation of nanostructures on electrode. Moreover, the entire structures can be reliably electrochemically interrogated. Typically, metal nanostructures (e.g., Pd or Au) can be constructed on the electrode surface by plating at a larger negative potential. A controlled nanostructure can be generated by employing an appropriate electrolyte and voltage. Kelley’s group prepared numerous of nanostructured electrodes and demonstrated their applications in detection of nucleic acid [67,68,69,70]. For example, in 2009, Yang* et al.* [67] prepared a nanostructured electrode for miRNAs detection by plating gold on the silicon surface (Figure 9). A layer of SiO_2_ was deposited on the gold-plating electrode surface to open an aperture of 500 nm. Then, palladium was electrodeposited in the aperture to generate highly nanostructured microelectrodes (NMEs). The resulting PNA/RNA complexation was assayed with the Ru^III^ redox reporter system. Moreover, Ru^III^ can be regenerated chemically by ferricyanide after its electrochemical reduction, thus amplifying the electrochemical signal. Consequently, a detection limit of 10 aM was achieved. Furthermore, Fang* et al.* [70] reported a sensing platform consisting of three-dimensional gold nanowires (Figure 10). The electrode was manufactured by electroless deposition of gold within the polycarbonate membranes. After oxygen plasma etching for 150 s, gold nanowires with a length of 200 nm and a diameter of 10 nm were exposed from the membrane surface. The PNA/RNA hybridization was also detected using the electrocatalytic Ru^III^/Fe^III^ reporter system. The method was able to specifically determine miRNAs down to 100 fM.

### 2.7. Magnetic Beads

Bead-based hybridization has the theoretical advantage that it might more closely approximate hybridization in solution [71]. Magnetic beads enable easy separation and washing steps. Even before the term “nanotechnology” was popular, iron oxide (Fe_3_O_4_) nanoparticles were used to magnetically isolate and purify proteins, DNA, viruses and even whole mammalian cells [72]. Recently, Bettazzi* et al.* [73] suggested the electrochemical detection of miRNAs using paramagnetic beads capped with biotinylated DNA capture probes. The miRNAs extracted from the cell sample were biotinylated and then hybridized with the CPs on the magnetic bead. After incubation with SA-ALP and washing with the buffer solution, the beads were exposed to the α-naphthyl-phosphate substrate. Then, the current from the enzymatic product was electrochemically measured with a compact microfluidic device. This method enabled multiplexed analysis with a low detection limit (7 pM). Similarly, Erdem* et al.* [74] also reported the ALP-amplified voltammetric detection of miRNAs with magnetic beads assay on multi-channel screen-printed array of electrodes.

**Figure 11:**
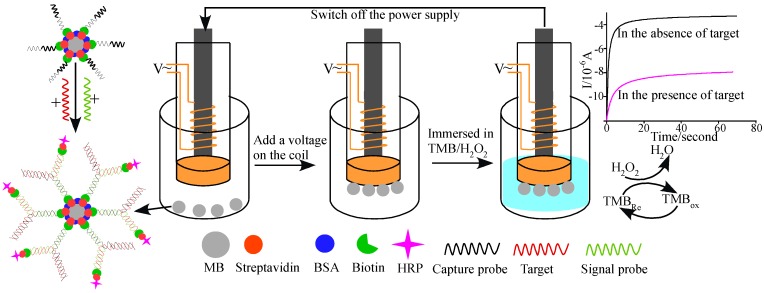
The principle of the magnetic-controllable electrochemical RNA biosensor. Reprinted with permission from [75]. Copyright 2013 Elsevier.

Wang* et al.* [75] developed a magnetic-controllable electrochemical RNA biosensor for the detection of oral cancer-related miRNAs based on a “junction-probe” strategy on a home-made electrically magnetic-controllable gold electrode (Figure 11). In the work, the biotinylated CPs were modified on the surface of streptavidin-anchored magnetic beads. The signal probes were derivatized with biotin tags at the two ends. With the addition of target miRNAs, the CPs hybridized with both miRNAs and signal probes to form the ternary “Y” junction structures on the surface of magnetic beads. After capture of SA-HRP by the biotinylated signal probes, the hybrid-attached magnetic beads were anchored on the surface of the electrically magnetic-controllable working electrode by endowing a voltage on the electric coil. The resulting HRP-tagged magnetic beads can catalyze the H_2_O_2_-mediated oxidation of TMB. As a result, miRNAs at the concentration below 0.22 aM can be detected readily with a recovery of 93%–108%. The linear range of this method was 1 aM~10 fM. Additionally, Bartosik* et al.* [76] also described the detection of miRNAs using magnetic beads. In the work, miRNAs were pre-labeled with electroactive Os(VI)bipy complex on the ribose of the 3′-end of miRNA. The Os(VI)bipy-labeled miRNAs can be captured by the complementary capture probe-capped magnetic beads and then thermally released and electrochemically detected at hanging mercury drop electrode.

**Figure 12:**
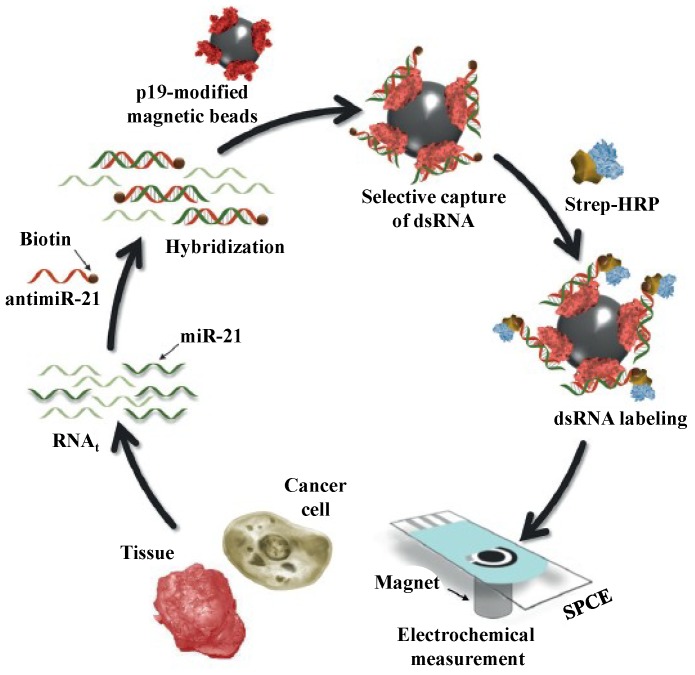
Schematic representation of the p19-based amperometric magnetosensor designed for the determination of miR-21. The components of the magnetosensor are not drawn to scale. Reprinted with permission from [77]. Copyright 2014 John Wiley and Sons.

Very recently, Campuzano* et al.* [77] developed an electrochemical magnetosensor for direct determination of miRNAs in RNAt raw sample on commercial screen-printed electrodes (Figure 12). In this work, p19 protein was immobilized onto chitin-functionalized magnetic beads (chitin-magnetic beads) for the capture of the miRNAs/anti-miRNAs duplex. 

SA-HRP was anchored onto the magnetic beads through the streptavidin-biotin interaction. The resulting magnetic beads were then magnetically captured by the screen-printed carbon electrodes (SPCEs). By measuring the catalytic amperometric current upon the addition of H_2_O_2_ and hydroquinone, the method showed linearity between 0.14 and 10.0 nM with a detection limit of 0.04 nM.

## 3. Conclusions/Outlook

MiRNAs play significant functions in numbers of developmental and physiological processes. They are also regarded as promising biomarkers and therapeutic targets in cancer treatment. Recently, the short length, low expression and high sequence similarity of miRNAs arouse the creation of new techniques for their sensitive and selective detection in complex samples. Nanomaterials-based electrochemical sensing strategies offer numerous advantages over traditional molecular diagnostic, such as signal amplification, improved sensitivity as well as simplicity, and versatile sensing scheme that can be tailored to a desired target. Since the first electrochemical miRNAs biosensor was reported in 2006, considerable efforts have been made to enhance the sensitivity for miRNAs detection by utilizing the unique chemical and physical properties of nanostructures. This work reviewed the progress in the development of electrochemical miRNAs biosensors using functional nanoscaffolds of novel nanomaterials, such as metal and metal oxide nanoparticles, CNTs, QDs, nanostructured electrodes and magnetic beads. Although there are still limitations for their practical use as regular methods in clinical diagnostic and prognostic, the advances in nanoscience and nanotechnology promise a better future for the biosensor industries.
